# Draft Genome Sequence of *Bacillus murimartini* LMG 21005^T^, an Alkalitolerant Bacterium Isolated from a Church Wall Mural in Germany

**DOI:** 10.1128/genomeA.01229-15

**Published:** 2015-10-22

**Authors:** Jie-ping Wang, Bo Liu, Guo-hong Liu, Rong-feng Xiao, Xue-fang Zheng, Huai Shi, Ci-bin Ge

**Affiliations:** Agricultural Bio-Resources Research Institute, Fujian Academy of Agricultural Sciences, Fuzhou, Fujian, China

## Abstract

*Bacillus murimartini* LMG 21005^T^ is a Gram-positive, spore-forming, and alkalitolerant bacterium isolated from a church wall mural. Here, we report the 4.17-Mb genome sequence of *B. murimartini* LMG 21005^T^, which will accelerate the application of this alkalitolerant bacterium and provide useful information for genomic taxonomy and phylogenomics of *Bacillus*-like bacteria.

## GENOME ANNOUNCEMENT

The type strain LMG 21005^T^ of *Bacillus murimartini* was isolated from a church wall mural in Germany and identified as a novel alkalitolerant member of the *Bacillus* rRNA group 6 (1). *B. murimartini* LMG 21005^T^ showed 98.8% similarity with the closest described neighbor, *Bacillus gibsonii* DSM 8722^T^ (2). The optimal growth of *B. murimartini* LMG 21005^T^ was achieved at 15°C to 20°C and pH 8.5; it was not salt tolerant (up to 4% NaCl) (1). Notably, there is no other information about *B. murimartini* except its taxonomical description so far. Given no available genomic information of *B. murimartini*, its type strain LMG 21005^T^ was selected as one of the research objects in our “genome sequencing project for genomic taxonomy and phylogenomics of *Bacillus*-like bacteria.” Here, we presented the high-quality draft genome sequence of *B. murimartini* LMG 21005^T^.

The genome sequencing of *B. murimartini* LMG 21005^T^ was performed via the Illumina HiSeq 2500 system. Two DNA libraries with insert sizes of 500 and 5,000 bp were constructed and sequenced. After filtering of the 1.33-Gb raw data, the 1.27-Gb clean data were obtained, providing approximately 200-fold coverage. The reads were assembled via the SOAPdenovo software version 1.05 (3), using a key parameter K setting at 71. Through the data assembly, 13 scaffolds with total length 4,169,138 bp were obtained, and the scaffold *N*_50_ was 1,047,322 bp. The average length of the scaffolds was 320,702 bp, and the longest and shortest scaffolds were 1,178,572 and 508 bp, respectively. A total of 89.15% clean reads were aligned back to the genome, which covered 99.95% of the sequence,

The annotation of the genome was performed using the NCBI Prokaryotic Genomes Automatic Annotation Pipeline (PGAAP) (http://www.ncbi.nlm.nih.gov/genomes/static/Pipeline.html) utilizing GeneMark, Glimmer, and tRNAscan-SE tools (4). A total of 4,212 genes were predicted, including 4,120 coding sequences (CDS), 14 pseudogenes, 71 tRNAs, and 6 rRNA genes. There were 3,162 and 2,422 genes assigned to COG and KEGG databases, respectively. The average DNA G+C content was 43.40%, with a significant difference from the value 39.6 mol% acquired by or high-performance liquid chromatography (HPLC) determination (1).

### Nucleotide sequence accession numbers.

This whole-genome shotgun project has been deposited at DDBJ/EMBL/GenBank under the accession no. LGUH00000000. The version described in this paper is version LGUH01000000.
